# The Association Between HbA1c and Other Biomarkers With the Prevalence and Severity of Diabetic Retinopathy

**DOI:** 10.7759/cureus.12520

**Published:** 2021-01-06

**Authors:** Noura M Almutairi, Shoroog Alahmadi, Mona Alharbi, Sarah Gotah, Majed Alharbi

**Affiliations:** 1 College of Medicine, Taibah University, Medina, SAU

**Keywords:** diabetes mellitus, diabetic retinopathy, hba1c, saudi arabia

## Abstract

Introduction: Diabetes mellitus (DM) is considered to be a significant public health problem globally. According to the American Society of Retina Specialists, diabetic retinopathy (DR) is a complication of diabetes that causes damage to the blood vessels of the retina. In a Japanese study showed that HbA1c and fasting blood glucose values can be considered as predictors for the future development of DR. Thus, this study aimed to determine the prevalence and severity of DR among diabetic patients in Medina, Saudi Arabia, and to assess its relationship with HbA1c and other biomarkers.

Methods: This cross-sectional study was conducted at Prince Abdulaziz Bin Majed Diabetes Center and Charitable Healthcare Society in Medina, Saudi Arabia, in July 2018. The data were collected from direct interview questionnaires that were administered to 130 randomly selected diabetic subjects. The subjects’ demographic information, eye disease history, medical backgrounds, and laboratory biomarkers were noted. Fundus examinations using a slit lamp were conducted by ophthalmic physicians to diagnose and grade DR. The data were analyzed using Statistical Package for Social Sciences (SPSS) (IBM Corp., Armonk, NY, USA).

Results: All 130 diabetic patients were examined for DR. DR was prevalent among 35 (26.9%) of the participants. According to the DR severity grading, 95 (73.1%) had no apparent DR, 11 (8.5%) had mild non-proliferative diabetic retinopathy (NPDR), 11 (8.5%) had moderate NPDR, 11 (8.5%) had severe NPDR, and only two (1.5%) had proliferative diabetic retinopathy (PDR). There was a significant association between the development of DR and HbA1c levels (p = 0.040). The duration of DM was also determined to be a significant risk factor for DR (p = 0.001). No other factors were found to have a significant association with DR.

Conclusion: Around one-third of the participants in our study had DR. HbA1c levels and duration of DM were established as important risk factors for DR. Screening is necessary, even in patients with good HbA1c levels, to avoid the late presentation of severe DR and to prevent blindness.

## Introduction

Diabetes mellitus (DM) is considered to be a significant public health problem globally as its incidence has increased dramatically in recent years [1]. In addition to the mordant effect of DM on individuals, it also places a heavy economic burden on countries [2]. The World Health Organization defines DM as a fasting plasma glucose ≥ 7.0 mmol/l (126 mg/dl) or two-hour plasma glucose ≥ 11.1 mmol/l (200 mg/dl) [3]. The symptoms of DM are usually less marked in the early stages, so most patients are diagnosed when they already have complications [4,5]. Chronic hyperglycemia that results from insufficient insulin secretion and/or the inability to use insulin effectively can lead to serious life-threatening conditions, including cardiovascular problems, retinopathy, nephropathy, and neuropathy [6]. According to the American Society of Retina Specialists, diabetic retinopathy (DR) is a complication of diabetes that causes damage to the blood vessels of the retina- the light-sensitive tissue that lines the back part of the eye, allowing you to see fine details [7].

The International Diabetes Federation has indicated that the prevalence of DM in Saudi Arabia (SA) reached 24% in 2013, and the country is ranked as having the seventh-highest prevalence of DM worldwide [8]. The prevalence of DR among SA cities varies from 11.3% to 36.4%, with the highest prevalence in Abha and the lowest in the Aseer region [9]. Many risk factors can accelerate the progression of DR in diabetic patients. Modifiable risk factors include blood glucose control, lipid profile, blood pressure, and smoking. Non-modifiable risk factors are patient age, the duration of DM, a genetic predisposition, and ethnicity [5,6].

In a Japanese study, Nakagami et al, [1] showed that hemoglobin A1c (HbA1c) and fasting blood glucose values can be considered as predictors for the future development of DR. A population-based cross-sectional survey conducted in Jazan, SA, reported that 71% of severe visual impairment cases were due to cataract, followed by uncorrected refractive errors in 13% of the subjects, and DR in 5% [10]. In contrast, another study conducted in the north of SA showed that the refractive errors rate was 36%, the cataract rate was 29.1%, and the rate of DR was 20.9% [11]. Regular eye screening is therefore essential to detect DR in its early stages and to prevent blindness [2,4].

This study aimed to address most of the limitations of the previous studies conducted in Medina, such as data being collected from only one diabetes center and an overestimation of its prevalence by supposing that diabetic patients with blindness and cataract had DR as the primary cause without studying the association between DR and other biomarkers such as total cholesterol, triglycerides, and creatinine [12,13].

## Materials and methods

This was a cross-sectional study conducted in Prince Abdulaziz Bin Majed (PABM) Diabetes Center and Charitable Healthcare Society (CHCS), Medina, Saudi Arabia.

The sample size comprised 130 known diabetic subjects (76 females and 54 males) who visited the PABM Diabetes Center and CHCS during July 2018. The subjects were selected randomly. The inclusion criteria were ≥ 15 years of age, visiting the PABM Diabetes Center and CHCS for follow up, and previously diagnosed with type 1 or type 2 DM. Diabetic patients younger than 15 years, patients with missing data, and patients with gestational diabetes were excluded.

The data were collected via direct interviews with the participants. The interviews were conducted by researchers who filled out questionnaires that required information about demographic and clinical parameters. The demographic parameters included gender, age, nationality, place of residence, education level, occupation, marital status, financial status, smoking status, and family history of DM or eye disease. The clinical parameters were the type of DM, the duration of DM, the type of diabetes medication, any complications of DM, hypertension, renal impairment, coronary artery disease (CAD), the use of antihypertensive or antihyperlipidemic agents, the time of the last visit for an eye examination, and receiving focal laser, pan-retinal laser, anti-vascular endothelial growth factor injections, or eye surgery.

The study protocol was approved by the Institutional Review Board at King Fahad Hospital, Medina, Saudi Arabia (approval IRB-TU-0013). Informed consent was obtained from all subjects. The last HbA1c, body mass index (BMI), triglycerides, total cholesterol, and serum creatinine results were taken from each subject’s file. We determined whether the diabetic patients had a good or poor control of their DM by referring to the American Diabetes Association guidelines where poor control of DM is defined as an HbA1c level ≥ 7.0% (53 mmol/mol) [14]. The National Institute for Clinical Excellence guidelines define healthy weight as BMI 18.5 to 24.9 kg/m^2^, overweight as BMI 25-29.9 kg/m^2^, and obesity as BMI ≥ 30 kg/m^2^ [15]. Triglycerides with level of ≥ 2.26 mmol/L was considered high, total cholesterol was high if ≥ 6.2 mmol/L [16]. Visual acuity was assessed using a standardized chart (Snellen E chart) and intraocular pressure using Goldman applanation tonometer and a non-contact tonometer (Reichert Technologies Inc, Depew, NY, USA). After applying mydriatic eye drops (Tropicamide 1%, Alcon-Couvreur, Puurs, Belgium), an examination of the fundus was conducted by ophthalmologists using a slit lamp (Reichert Technologies) to detect the presence of DR and its severity. The severity of DR is classified by the International Clinical Diabetic Retinopathy Disease Severity Scale into five stages (no apparent DR, mild non-proliferative diabetic retinopathy (NPDR), moderate NPDR, severe NPDR, and proliferative diabetic retinopathy (PDR)) [17].

Data were analyzed using Statistical Package for Social Sciences (SPSS) version 25 (IBM Corp., Armonk, NY, USA) software to study the relationship between the HbA1c of the diabetic patients, which reflects their diabetes control, and other studied factors and the presence of DR and its severity. The chi-square test and logistic regression test were applied. A p-value < 0.05 was considered statistically significant. Descriptive statistics like mean, standard deviation (SD), and the independent-one sample t-test were used to determine the significance of the differences between the means of the studied factors. 

## Results

**Figure 1:**
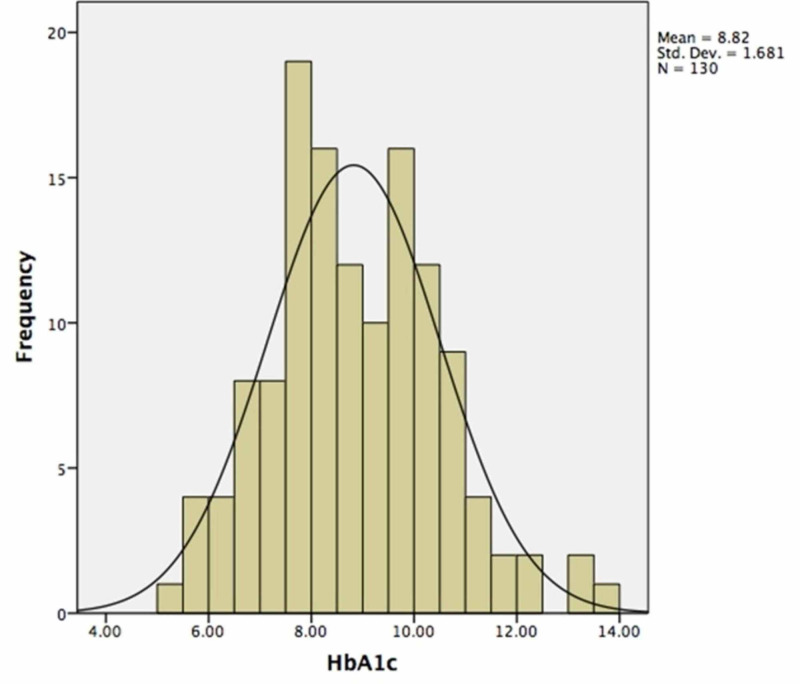
Histogram showing the distribution of HbA1c levels among the study participants

In total, 130 diabetic patients who attended the ophthalmic clinics in July 2018 were included in our study. Of these, 111 had type 2 DM and 19 had type 1 DM. Most of the patients (76; 58.5%) were female with a mean age 49.8 ± 16.2 years, and 54 (41.5%) were male with a mean age of 54.1 ± 12.4 years. The mean age (51.5 ± 14.8 years) showed no statistically significant difference when compared to gender (p = 0.094). The mean duration of DM was 11.5 ± 8.8 years. When matched to the presence or absence of DR (15.9 ± 8.3 years and 10 ± 8.5 years, respectively), a statistically significant difference (p = 0.001) was evident. As shown in Figure 1, the mean HbA1c among the patients was 8.8 ± 1.7. It was less than 7% in 17 patients (13.1%) and ≥ 7% in 113 patients (87%). There was no significant difference between the mean HbA1c of the male and female patients. The prevalence of DR among the participants was 35 (26.9%).

As shown in Table 1, the chi-square test, which was used to assess the relationship between DR and the studied factors, showed that the relationship between the presence of DR and the duration of DM was significant (p = 0.001), as was the relationship between DR presence and HbA1c levels (p = 0.036). However, there was no significant association between DR and age, level of education, work status, financial status, follow-up status, and BMI. According to the DR severity grading, 95 (73.1%) of the patients had no apparent DR, 11 (8.5%) had mild NPDR, 11 (8.5%) had moderate NPDR, 11 (8.5%) had severe NPDR, and only two (1.5%) had PDR. There was a significant association between the severity of DR and the duration of DM (p = 0.021) and the severity of DR and the presence of a family history of DM (p = 0.036), but there was no significant relationship between HbA1c and the severity of DR (p = 0.293). 

**Table 1:** Chi-square test showing the association between diabetic retinopathy and the studied factors. HbA1c: hemoglobin A1c, DM: diabetes mellitus

Variable		Total	Diabetic retinopathy		p-value
			Yes, n (%)	No, n (%)	
Duration of DM	1–5 years	46	6 (13)	40 (87)	0.001
	6–10 years	27	4 (14.8)	23 (85.2)	
	≥ 11 years	57	25 (43.9)	32 (56.1)	
HbA1c level	< 7%	17	1 (5.9)	16 (94.1)	0.036
	≥ 7%	113	34 (30.1)	79 (69.9)	
Age	15–29 years	15	1 (6.7)	14 (93.3)	0.255
	30–49 years	27	7 (25.9)	20 (74.1)	
	50–59 years	46	13 (28.3)	33 (71.7)	
	≥ 60 years	42	14 (33.3)	28 (66.7)	
Level of education	Illiterate	21	7 (33.3)	14 (66.7)	0.227
	Can read and write only	15	5 (33.3)	10 (66.7)	
	Primary	27	9 (33.3)	18 (66.7)	
	Intermediate	22	4 (18.2)	18 (81.8)	
	High school	18	1 (5.6)	17 (94.4)	
	University	27	9 (33.3)	18 (66.7)	
Work status	Housewife	46	15 (32.6)	31 (67.4)	0.411
	Worker	34	8 (23.5)	26(76.5)	
	Non-worker	21	4 (19)	17 (81)	
	Student	5	0 (0)	5 (100)	
	Retired	24	8 (33.3)	16 (66.7)	
Financial status	Low	26	5 (19.2)	21 (80.8)	0.202
	Intermediate	99	30 (30.3)	69 (69.7)	
	High	5	0 (0)	5 (100)	
Follow-up status	First time	6	0 (0)	6 (100)	0.256
	Irregular	37	9 (24.3)	28 (75.7)	
	Regular	87	26 (29.9)	61 (70.1)	
BMI	Normal	42	9 (21.4)	33 (78.6)	0.312
	Overweight	28	6 (21.4)	22 (78.6)	
	Obese	60	20 (33.3)	40 (66.7)	

In Table 2, logistic regression showed that the duration of DM was significant as a risk factor for DR with a p-value = 0.001. HbA1c was similarly significant with a p-value = 0.040. Otherwise, no significant association was observed between DR and the other studied factors (gender, family history of DM, smoking, hypertension, CAD, renal impairment, cholesterol levels, triglycerides, and serum creatinine).

**Table 2:** The relationship between diabetic retinopathy and the studied factors after adjusting for confounding effects using logistic regression HbA1c: hemoglobin A1c, DM: diabetes mellitus

Variable		Total	Diabetic retinopathy		OR	95% CI	p-value
			Yes, n (%)	No, n (%)			
Duration of DM	1–5 years	46	6 (13)	40 (87)	-	-	-
	6–10 years	27	4 (14.8)	23 (85.2)	1.2	0.296 -4.541	0.832
	≥ 11 years	57	25 (43.9)	32 (56.1)	5.2	1.907 -14.228	0.001
HbA1c level	< 7%	17	1 (5.9)	16 (94.1)	-	-	-
	≥ 7%	113	34 (30.1)	79 (69.9)	6.9	0.878 - 54.002	0.040
Gender	Male	54	13 (24.1)	41 (75.9)	-	-	-
	Female	76	22 (28.9)	54 (71.1)	1.3	0.579 -2.851	0.537
Family history of DM	Yes	86	24 (27.9)	62 (72.1)	0.9	0.376 -1.973	0.724
	No	44	11 (25)	33 (75)	-	-	-
Smoking	Non-smoker	118	33 (28.0)	85 (72.0)	0.5	0.107 -2.477	0.400
	Smoker	12	2 (16.7)	10 (83.3)	-	-	-
History of hypertension	Yes	66	21 (31.8)	45 (68.2)	1.7	0.759 -3.662	0.203
	No	64	14 (21.9)	50 (78.1)	-	-	-
History of coronary artery disease	Yes	21	8 (38.1)	13 (61.9)	1.6	0.572 -4.347	0.415
	No	109	27 (24.8)	82 (75.2)	-	-	-
History of renal impairment	Yes	9	3 (33.3)	6 (66.7)	1.4	0.328 -5.890	0.701
	No	121	32 (26.4)	89 (73.6)	-	-	-
Total cholesterol	Normal	119	33 (27.7)	86 (72.3)	-	-	-
	High	11	2 (18.2)	9 (81.8)	0.6	0.119 -2.823	0.726
Triglycerides	Normal	93	26 (28)	67 (72)	-	-	-
	High	37	9 (24.3)	28 (75.7)	0.8	0.345 -1.991	0.673
Serum creatinine	Normal	120	32 (26.7)	88 (73.7)	-	-	-
	High	10	3 (30)	7 (70)	1.2	0.287 -4.836	0.819

## Discussion

In this study, the prevalence of DR among the patients with DM was 26.9%. In previous studies conducted in SA, the prevalence was 36.1%, 27.8%, 36.4%, and 14.8% in Medina [12], Jazan [10], Abha [9], and Riyadh [18], respectively. The studies that were conducted in Medina and Abha reported higher percentages due to the use of a fundus camera in addition to a slit lamp for diagnosis and grading. However, the study in Jazan showed a similar prevalence of DR to that in our study because similar tools were used for diagnosis. Globally, the prevalence of DR varies from 15% to 45% [19,20]. According to the DR severity grading, 73.1% of the patients in our study had no apparent DR, 8.5% had mild NPDR, 8.5% had moderate NPDR, 8.5% had severe NPDR, and 1.5% had PDR. Hajar et al. [10] found that 68.1% of the diabetic patients in their study had no apparent retinopathy, 18% had mild NPDR, 5.3% had moderate NPDR, 3.5% had severe NPDR, and 1.1% had PDR.

This study found a significant association between the development of DR and HbA1c levels (p = 0.040). However, there was no statistically significant relationship between HbA1c and the severity of DR (p = 0.293). The potential for diabetic patients to develop DR when their HbA1c ≥ 7% was 6.9 times greater than those with an HbA1c < 7%. The mean level of HbA1c among our study participants was 8.9 ± 1.7 with no statistically significant difference between the means of the males and females. In contrast, El-Bab et al. [12] reported that the mean HbA1c levels were significantly higher among the males than the females in their study. Some studies have shown a significant relationship between HbA1c and DR [13,19] although a significant association has also been demonstrated between HbA1c and the severity of DR [12]. In a cross-sectional study conducted in Abha, SA, HbA1c levels related to the presence of DR significantly in the univariable analysis but was insignificant in the multiple logistic regression [9]. In addition, a cross-sectional study found that the potential to develop DR in patients with an HbA1c ≥ 7% was increased 17.5 times, which was significantly different from those with good control [13]. Another study reported that patients with an HbA1c ≥ 7% were 1.9 times more likely to have DR than those with an HbA1c < 7% [20]. These high percentages of diabetic patients with elevated HbA1c levels indicate clearly that improving diabetes management in primary healthcare centers through the implementation of several key strategies is needed to decrease the prevalence and slow the progression of DR. Furthermore, increasing patient awareness of the consequences of poor control of DM is necessary.

In this study, which comprised 76 females and 54 males, the mean age of the females was 49.8 ± 16.2 years while the mean age of the males was 54.1 ± 12.4 years. When compared to gender, there were no statistically significant differences in mean age (p = 0.094). However, we found that the female patients in our study were 1.285 times more likely to have DR than the male patients. When comparing gender and HbA1c levels, 59% of the patients with an HbA1c < 7% were female and 41% were male (10:7 female:male). On the other hand, 58% of those with an HbA1c ≥ 7 were female and 42% were male (66:47 female:male). In terms of the severity of DR and gender, the female-to-male ratios were 1.3, 1.8, 1.8, 1.8, and 1 in the DR stages 1, 2, 3, 4, and 5, respectively. However, there was no statistically significant difference between DR and gender (p = 0.537). These findings are similar to studies by Valizadeh et al. [19] and Badawi et al. [13], which showed that there was no significant association between developing DR and gender.

With respect to DR and the duration of DM, the mean duration of DM in our study was 11.5 ± 8.8 years. When matched to the presence or absence of DR (15.9 ± 8.3 years and 10 ± 8.5 years, respectively), there was a statistically significant difference (p = 0.001). We found that patients with a DM duration ≥ 11 years were 5.2 times more likely to develop DR, while those with a duration of six to 10 years were 1.2 times to develop DR when compared with patients with a duration of one to five years. We also found that the duration of DM was significantly associated with both developing DR (p = 0.001) and the severity of DR (p = 0.021). The study by Badawi et al. [13] similarly showed that an increased duration of DM was associated with a higher risk of DR. In the study by Valizadeh et al. [19], no significant association was established between increased DM duration and developing DR; however, the researchers found that DR was more likely to occur in those with an increased DM duration. The prevalence of DR among type 1 diabetic patients with a duration of ≤ five years increased from 6.1% to 62% among those who had had the disease for ≥ 10 years, while in type 2 diabetic patients, the prevalence increased from 10% to 50% over a similar duration [6].

The risk of DR was higher when considering the moderating effects of HbA1c levels and DM duration. The main effects of HbA1c levels and duration of DM explained a 12.9% variance in DR, which was significant. The interaction between HbA1c levels and DM duration accounted for an additional significant 2.2% variance.

The prevalence of obese patients with DR in our study was 33.3%, and they had a 1.6 times increased risk of DR compared to the participants with a normal BMI, which was statistically insignificant. Furthermore, 21.4% of the overweight participants had DR, demonstrating a 0.9 times increased risk of DR compared to subjects with a normal BMI. This was also statistically insignificant. These results were similar to those of a study conducted in Medina, Saudi Arabia, which showed that obese participants were at a 1.5 times higher risk of developing DR compared to the controls with a normal BMI [13]. Likewise, this was found to be statistically insignificant.

To strengthen our study, some biochemical parameters were included to observe the relationship between certain parameters and the presence or absence of DR and the severity of DR. The statistical analysis showed an insignificant relationship between DR and high levels of cholesterol and triglycerides (p = 0.726 and 0.673, respectively). A chi-square test showed an insignificant relationship between cholesterol levels and the severity of DR (p = 0.621) and an insignificant association between triglycerides and the severity of DR (p = 0.509). Cetin et al. [21] showed that total cholesterol and triglyceride levels were not significantly associated with the presence and severity of DR. Contrarily, a study of type 2 diabetic patients found a significant association between total cholesterol and triglycerides and the development of DR [22]. A study in Qassem, SA, showed a statistically significant relationship between dyslipidemia and PDR. It can therefore be inferred that, by controlling blood glucose and/or the administration of lipid-lowering agents for type 2 diabetics, visual impairment from DR can at least be delayed or prevented [23].

Retinopathy and nephropathy are long-term complications of DM, so we included creatinine as a study factor. However, it was found to be statistically insignificant in the presence of DR (p = 0.819), with the risk of developing DR increasing only 1.2-fold compared to subjects with a normal creatinine level. This result was similar to that of a study conducted in Iran. It also showed an insignificant relationship between creatinine levels and the severity of DR (with a p = 0.1) [24].

There were 76 (58.5%) females with a mean age of 49.8 ± 16.2 years and 54 (41.5%) males with a mean age of 54.1 ± 12.4 years. The men and women were matched for age, and no statistically significant differences were evident (p = 0.094). Furthermore, there was no significant relationship between age and severity of DR (p = 0.076) and no statically significant relationship between the age and the presence of DR in general (p = 0.255). Notwithstanding, it was observed that the number of patients with DR increased with age. The probable reason for this finding was not so much age itself as it was the duration of DM. The sample of the first group (15-29 years) was smaller than the other groups and that might explain the single case that was found to have DR. It was noticed that the patient in question had had uncontrolled type 1 DM for 15 years, which might be another factor that induced DR. This result correlates with those of other studies in which the difference was noticed although it was not statistically significant [19,25]. Nevertheless, in other studies, a significant relationship was found between DR and increasing age [9,12,13].

We found no significant relationship between educational level and DR prevalence, primarily because there was an equal number of DR participants with primary school and university level education. A probable reason for this is the difference in the definition of education and awareness, meaning that not every educated person is necessarily sufficiently aware of diabetes complications and the effects of DM on the eyes.

There was also no significant relationship between work status, financial status, and follow-up status as risk factors and DR. These results correlated with those of another study [19] but conflicted with others [6,13]. One study reported that married, educated, and physically active participants had a lower risk of developing DR [25].

We found that non-smokers were 0.5 times less likely to develop DR than smokers although there was no significant association between the development of DR and smoking. Our study included 12 (9.2%) smokers and 118 (90.8%) non-smokers. Similarly, a study conducted in Abha, SA, found that smoking is not a significant factor for the future development of DR [9]. These results were however at variance with those of other studies where smoking was determined to be significantly related to DR and, in fact, increased the likelihood of developing DR [12,13,26].

Hypertension was found to be significantly associated with the development of DR in previous studies and to play a major role in the progression of DR; monitoring and controlling hypertension can therefore slow the progression of DR [13,20]. In this study, 66 (50.8%) of the participants had been diagnosed with hypertension, and 21 (31.8%) of them had DR. Furthermore, we found that the hypertensive subjects were 1.667 times more likely to develop DR, but that was not sufficient to count hypertension as a risk factor for DR. Similar results have been found in other studies [9,13]. Given these conflicting findings, we believe that hypertension deserves further investigation, and special parameters are needed to study the control of blood pressure and its effects on DR.

In our study, CAD was not determined to be a significant risk factor for DR. Only 21 patients (16.2%) had a history of CAD, which was not sufficient for comparison or to establish its significance as a risk factor. A study in Medina also found no evidence of a significant role of CAD in DR prevalence [12]. 

We did not find a significant relationship between renal impairment and the presence of DR. In our study, nine (6.9%) patients out of 130 had renal impairment and only three (33.3%) of these had DR. In other studies, estimated glomerular filtration rate (eGFR) was measured and found to have a significant association with DR as a decrease in eGFR accelerated the progression of DR [20,27]. Other studies have further suggested that the presence of microalbuminuria is a strong biomarker to indicate the early development of DR in type 2 diabetic patient [28,29]. We therefore recommend combining eGFR and microalbuminuria as parameters of renal impairment measurement in association with the prevalence and severity of DR in future studies.

Some other conditions were recorded during the eye examinations of the patients in or study. Fifty (38.5%) of the studied participants had cataracts. This percentage was expected on account of a review article that was published about cataracts in diabetic patients. It composed that one of the earliest vision complications of diabetes caused by cataract. Diabetic patients were three to five times at higher risk of developing cataract compared to non-diabetic counterparts [30]. Moreover, 10 (8%) of the participants in our study had glaucoma and increased optic disc cupping, six (4.6%) had clinically significant macular edema, three (2.3%) had maculopathy, and two (1.5%) had papilledema. Additionally, there was one (0.8%) case each of vitreous hemorrhage, interstitial corneal opacity, non-arteritic anterior ischemic optic neuropathy, retinal detachment, retinitis pigmentosa and shallow anterior chamber.

Some of the limitations that we faced were beyond our control, such as time constraints and access to data which affected the sample size. Some data were self-reported, such as the presence of hypertension, renal impairment, and CAD, which could have resulted in inaccurate measurements due to the underestimation of the actual number of patients with these diseases.

## Conclusions

Around one-third (26.9%) of the participants in our study had DR. HbA1c levels and duration of DM were significantly associated with the development of DR among diabetic patients. A duration ≥ 11 years of DM was found to lead to a five-fold increased risk of the development of DR compared to a duration ≤ five years, regardless of the diabetes control. Screening by an ophthalmologist is essential for people with DM, even those with a controlled HbA1c, especially if they have had DM for a long time. Early detection is vital to avoid the late presentation of severe DR and to prevent blindness. 
